# The Contribution of Co-signaling Pathways to Anti-malarial T Cell Immunity

**DOI:** 10.3389/fimmu.2018.02926

**Published:** 2018-12-14

**Authors:** Rebecca Faleiro, Deshapriya S. Karunarathne, Joshua M. Horne-Debets, Michelle Wykes

**Affiliations:** QIMR Berghofer Medical Research Institute, Brisbane, QLD, Australia

**Keywords:** malaria, immunity, inhibitory receptors on T cells, stimulatory receptors on T cells, Immuno-therapy, chronic malaria, cerebral malaria, experimental cerebral malaria

## Abstract

*Plasmodium* spp., the causative agent of malaria, caused 212 million infections in 2016 with 445,000 deaths, mostly in children. Adults acquire enough immunity to prevent clinical symptoms but never develop sterile immunity. The only vaccine for malaria, RTS,S, shows promising protection of a limited duration against clinical malaria in infants but no significant protection against severe disease. There is now abundant evidence that T cell functions are inhibited during malaria, which may explain why vaccine are not efficacious. Studies have now clearly shown that T cell immunity against malaria is subdued by multiple the immune regulatory receptors, in particular, by programmed cell-death-1 (PD-1). Given there is an urgent need for an efficacious malarial treatment, compounded with growing drug resistance, a better understanding of malarial immunity is essential. This review will examine molecular signals that affect T cell-mediated immunity against malaria.

## Introduction

Malaria is a disease caused by parasites of the *Plasmodium spp*. of which there are six species that infect humans: *P. falciparum, P. vivax, P. ovale, P. malariae, P. knowlesi*, and *P. cynomolgi*. Malaria causes serious morbidity in large populations (1) but unlike many other infections, infected individuals do not become resistant to subsequent infections. While adults will develop protection from severe symptoms, chronic, and low grade infections remain a major threat to eradication efforts due to a vast number of carriers. Overall, while tremendous progress has been made in controlling malaria, no vaccine has been completely successful and as such new approaches are required.

Malaria parasites, introduced by mosquitos, first infect the liver and then blood. It is the blood-stage infection that causes the symptoms and lethality associated with malaria. Infants and children in endemic areas are highly susceptible to malaria during the first 5 years but slowly gain resistance to severe, life-threatening infections and then to clinical disease (1). However, sterile immunity is rarely attained (2). As such, there have been well over 100 clinical trials to develop a malaria vaccine and the only one to be approved for use is a pre-erythrocytic vaccine, RTS,S, which comprises of an immunogenic fragment of the circumsporozoite protein of *P. falciparum* with the hepatitis B surface antigen. In field trials, the vaccine showed 30–50% efficacy in the first year following vaccination and this dropped to only 16% in the fourth year, indicating that protective immunity is at some point compromised.

Optimal immune responses against infections require a balance between pro-inflammatory and regulatory immune responses. Pro-inflammatory responses drive protective immunity while regulatory responses control these immune response to prevent tissue damage and also prevent autoimmunity. Immunity against malaria requires a combination of antibodies and T cell responses. Recent research indicates that CD4^+^ T cells, which consist of several helper-subtypes that shape immune responses, play a much larger role in diminished malarial immunity than previously understood.

Original studies which transferred serum from malaria-protected adults to children, established that antibodies had a critical role in the clearance of parasites (3). However, mouse models have underpinned our understanding of T cell-immunity against malaria. Studies with *P. chabaudi*, which causes a rapidly resolving acute parasitemia followed by multiple recrudescent bouts over many months showed that CD4^+^ T cells are required to control malaria by their ability to help B cells (4, 5). The resultant antibodies promote parasite clearance by phagocytosis (6). Other studies with *P. chabaudi* showed that CD4^+^ T cells control the peak of parasitemia in the primary phase of acute blood stage infections (7, 8) via production of high levels of interferon-γ (IFN-γ) and tumor necrosis factor alpha (TNF-α) (9–11). Studies have also shown that IFN-γ and TNF-α cooperate to induce nitric oxide synthase expression in the spleen to control peak parasite burden (12). In contrast, *P. yoelii* YM, *P. yoelii* XL and *P. berghei* ANKA are severe, lethal infections with the last causing cerebral malaria (CM) as this parasite sequesters from the blood into deep tissues including the brain. Studies in mice revealed that CD8^+^ T cells sequester in the brain during cerebral malaria (13) and with early production of IFN-γ (14) were responsible for mortality. Similarly, CD8^+^ T cells mediate the loss of marginal metallophilic macrophages and damage to splenic architecture (15). However, several studies have now shown a role for these cells in controlling malaria (7, 16, 17) and more specifically their requirement to control chronic malaria (18) and for long-term sterile immunity (19). These studies suggest that vaccines for blood-stage malaria, would need to target multiple cell types including CD8^+^ T cells, which has not been undertaken.

### The Contribution of Co-signaling Pathways to Anti-malarial Immunity

**Figure 1:**
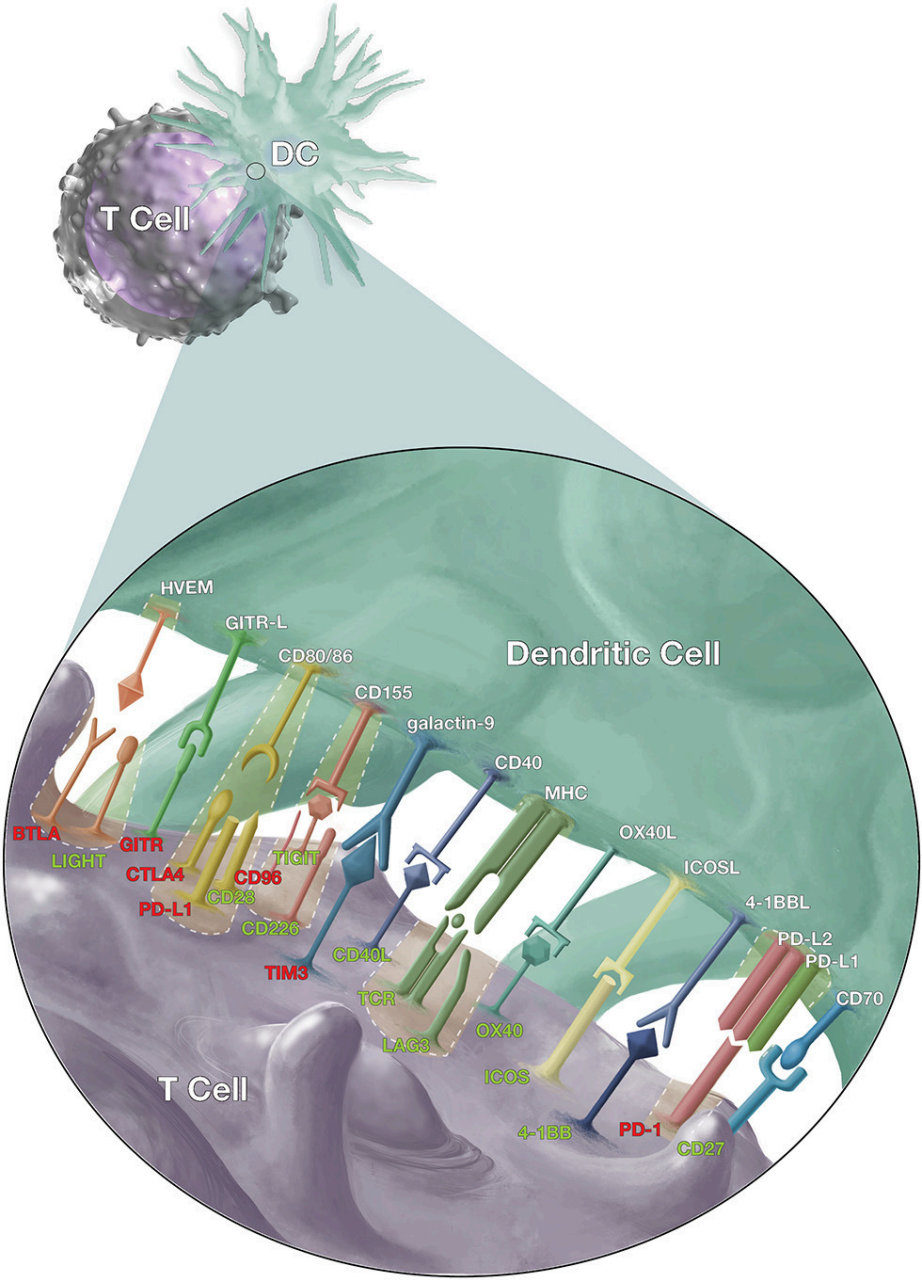
Co-stimulatory and co-inhibitory signals that modulate T cell responses. Dendritic cells (DCs) and T cells interact via several pathways to regulate T cell response against malaria, other pathogens and cancer cells. The T cell receptors (TCR) on antigen-specific T cells first recognize their cognate antigen via the major histocompatibility complex (MHC) molecules on antigen presenting cells. This is followed by the CD28/CD86 interaction, the “second signal,” and then a multitude of other signals to fine-tune the immune response. The red labels highlight inhibitory signals while the green show stimulatory signals and cases where inhibitory and stimulatory signals compete for the same ligand or receptor.

Signaling between antigen presenting cells [(APCs) including dendritic cells (DC), macrophages, and monocytes] and T cells (Figure 1) is a crucial element of adaptive T cell immunity which allows antigen-specific responses to be tightly regulated for effective protection against infections, while minimizing immune-mediated pathology. However, while many of these ligands and receptors are also expressed on other cells (e.g., B cells); in the interest of brevity, this review will focus on signaling between APCs and T cells.

While the interaction of the T cell receptor (TCR) with peptide-loaded major histocompatibility complexes (MHC) on APCs is essential for antigen-specific T cell activation, co-stimulatory (activating), and co-inhibitory (suppressive) molecules determine the magnitude and type of T cell responses. Furthermore, these interactions also co-signal APC to modulate their functions. The requirement for co-stimulation in T cell activation was realized with the discovery of the function of CD28, the canonical co-stimulatory receptor (20, 21). Since then, a large number of co-stimulatory or co-inhibitory receptors, ligands, and counter-receptors have been discovered and studied.

The study of receptor and ligands in the context of infections, cancers and autoimmunity, has revealed that the modulation of co-signaling pathways (also known as “immune checkpoints”) underpins the pathogenesis of several diseases. The balance between co-stimulatory and co-inhibitory receptor activation can have a profound effect on disease progression. Recent advances in the understanding of which immune checkpoints influence disease has led to the development of specific targeted immunotherapies that modulate immune functions to alleviate disease. Most immunotherapies utilize antibodies to modulate immunity and in particular, antibodies against the co-inhibitory receptors cytotoxic T-lymphocyte associated antigen-4 (CTLA-4) and PD-1 have been approved by the FDA for treatment of several cancers due to their exceptional efficacy.

Effective immunity against intracellular pathogens requires the development of an optimal T cell response which shows rapid proliferative potential, low apoptosis and poly-functionality (22). During acute infections, optimal functioning T cells clear the pathogen, eventually leading to development of robust memory T cells which have the ability to mount rapid recall response and re-establish poly-functional effector mechanisms upon antigen re-exposure (23). However, some diseases develop “T cell exhaustion,” defined by poor effector function, sustained expression of inhibitory receptors (discussed later in detail), poor recall responses and a transcriptional state distinct from that of functional effector or memory T cells (23). There is increasing evidence that these inhibitory receptors affect malarial immunity. As such, the remainder of the review will look at each inhibitory / stimulatory pathway individually and discuss their roles in regulating immunity to *Plasmodium* infection.

### Programmed Cell Death Protein-1

PD-1 (CD279) is a member of the extended CD28/CTLA-4 family of T cell regulators (24). PD-1 is expressed on T cells, B cells, natural killer, dendritic cells (DCs) and activated monocytes. The engagement of PD-1 by its ligands, PD-L1 (CD274), and PD-L2 (CD273), normally inhibits T cell functions to induce tolerance and to control the expansion and function of foreign antigen-specific T cell-responses (25–27). PD-1 is not expressed by resting T cells but is induced upon activation (28). PD-1 expression on T cells is up-regulated within 24 h of stimulation and the effects of PD-1 ligation can be seen within a few hours (29). PD-1 needs to be engaged simultaneously with TCR signals to trigger an inhibitory signal (30). PD-1 preserves exhausted T cell populations from overstimulation, excessive proliferation, and terminal differentiation (31). In general, interactions between PD-1 on T cells and its ligand, PD-L1, control the induction and maintenance of peripheral T cell tolerance during normal immune responses and negatively regulate the proliferation and the cytokine production by T cells (32).

Field studies in malaria-endemic areas have shown increased expression of PD-1 on T cells in malaria-infected individuals compared to control subjects, thus implicating PD-1 in immune evasion (33, 34). Significantly, flow cytometric analysis and automated multivariate clustering, has revealed more frequent expression of CTLA-4 or PD-1 on CD4^+^ T cells from children with complicated malaria compared to uncomplicated malaria (35). PD-1-deficient mice (PD-1KO) rapidly cleared chronic *P. chabaudi* malaria and developed sterile immunity unlike infections in wild type mice (18). Subsequent studies in mice showed that PD-1 also mediated loss of long-term protection against malaria (19). These studies clearly show PD-1 contributes to the pathogenesis of malaria.

PD-L1 and PD-L2, the ligands for PD-1, are expressed on a variety of cells, and their expression on DCs can down-regulate immune responses by T cells (27). Significantly, blockade of PD-L1 signals by antibody, during malaria contributed to improved immunity (33). Furthermore, combined blockade of PD-L1 and Lymphocyte-activation gene 3 (LAG3) immune inhibitory molecules accelerated clearance of non-lethal *P. yoelii* blood-stage malaria by improving CD4^+^ T cell functions and increasing antibody titres (33). Similarly, blockade of PD-L1 during lethal *P. berghei*-induced experimental cerebral malaria (ECM) also enhanced T cell functions but resulted in an unfavorable outcome in this model, as improved T cell function promoted cerebral disease (36). These studies provide evidence that manipulating checkpoint proteins can improve immune responses.

Other studies of malaria using four mouse models revealed a novel regulatory function for PD-L2 (37). These studies showed that while PD-L1 expressed by DCs down-regulates T cells responses against malaria, PD-L2 protein expressed on DCs improves immune responses by inhibiting PD-L1–PD-1 interactions (37). A therapeutic role for PD-L2 was shown when multimeric form of PD-L2 given to mice infected with lethal malaria, was sufficient to clear the lethal infection and mediate survival from re-infections after several months, without additional treatment (37). Studies of healthy human volunteers before and after infection with experimental *P. falciparum* malaria, found that the expression of PD-L2 inversely correlated with the level of parasitaemia in each individual (37). Overall, this study highlighted the importance of PD-L2 expression for generating malarial immunity.

### CD28, CTLA-4, and CD80/CD86

CD28 is a co-stimulatory receptor of the immunoglobulin superfamily, which activates a variety of T cell-activation pathways such as nuclear factor-κB (NF-κB), nuclear factor of activated T cells (NFAT), BCL-XL and mammalian target of rapamycin (mTOR). These signals enhance functions associated with T cell activation such as IL-2 expression, proliferation, survival, and other effector mechanisms.

CD28 was found to be crucial for development of both polyclonal as well as specific antibody responses against malaria, as mice deficient in CD28 had a severe deficit in Ig-production by B cells by day 7 post-infection with *P. chabaudi* compared to WT mice (38). The effects of the CD28 deficit resulted in an inability of these mice to clear parasitemia to sub-patent levels or control re-infections. T cell proliferation was also severely compromised in CD28 deficient mice infected with *P. chabaudi*, although it was not determined whether CD28 directly influenced T cell help, B cell activation and antibody production, or both (38). Another study showed that in ${\text{J}}_{\text{H}}^{-/-}$ mice which lack B cells, a deficiency of CD28 resulted in diminished clearance of *P. chabaudi* parasitemia, demonstrating that CD28 is crucial to both humoral and cell-mediated immunity to malaria (39).

The ligands for CD28 (B7 molecules CD80 and CD86) are expressed by APCs (40). CD80 and CD86 can also bind the inhibitory receptor, CTLA-4 (CD152) which appears on T-cell following activation (41, 42). The mechanism of CTLA-4 function is complex with contradictory reports. CTLA-4 has a higher affinity for CD80 and CD86 than CD28 (43) and is believed to outcompete CD28 for binding to B7 ligands to regulate early events in T cell activation (44). CTLA-4 is also indicated to directly inhibit TCR signals, reduce IL-2 production and IL-2 receptor expression, and regulate cell cycle progression (44). It was also shown that CTLA-4 can capture B7 ligands from opposing cells by a process of trans-endocytosis resulting in impaired co-stimulation via CD28 (45). CD28 and CTLA-4 binding to CD80 and CD86 thus provide a balance between activation and inhibitory signals. Significantly, PD-L1 expressed by T cells also binds to CD80 on DCs to inhibit T cell activation (46, 47). Importantly, CD28 and PD-L1 on T cells compete for CD80 binding.

Studies using *P. chabaudi* malaria have revealed that antibody blockade of CD80 binding to CD28 did not significantly affect the clearance of parasitemia (48). In contrast, CD86 blockade or dual blockade of CD80 and CD86 resulted in the inability of mice to clear parasitemia to sub-patent levels (48). CD86 or CD80/CD86 blockade increased IFN-γ production while decreasing IL-4 production by *ex vivo* spleen cells (48). *P. chabaudi*-specific IgG1 antibody titres were also reduced by CD86 blockade while early IgG2a titres were increased (48). These effects were not observed with CD80 blockade alone, but were more drastic with dual blockade. This study, along with studies on the role of CD28, indicate that CD86 ligation of CD28 is essential for protective humoral immunity to malaria, while cell-mediated immunity also requires CD28 ligation.

Increased proportions of CD4^+^ T cells express CTLA-4, OX40, Glucocorticoid-induced TNFR family related gene (GITR), tumor necrosis factor alpha receptor type II (TNFRII), PD-1, LAG3, T-cell immunoglobulin and mucin-3 (TIM3) and CD69 during *P. vivax* (49, 50) and *P. falciparum* (51) malarias. The increased proportion of these cells did not correlate with parasite density in *P. vivax* infections, and did not persist after parasite clearance. However, simultaneous blockade of the CLTA-4, PD-1, and TIM3 signaling restores the cytokine production by antigen-specific cells (50). Significantly, expression of CTLA-4 by T cells is increased in children only during severe malaria (52).

Mouse studies have also shown that lethal malaria induces the production of high levels Transforming Growth Factor- β (TGF-β), which is associated with delayed and blunted IFN-γ and TNF-α responses, failure to clear parasites, and 100% mortality (53). Mechanistic studies showed that cross-linking surface CTLA-4 in cultures of spleen cells taken from mice infected with a lethal infection, induced TGF-β secretion (53). In contrast, blockade of CTLA-4 in mice was found to increase T cell activation and IFN-γ production, resulting in a lower peak parasitemia during non-lethal *P. yoelii* 17XNL infections (54) and could induce cross-species protection against *P. berghei* by inhibition of regulatory T cells (Treg) development (55). Similarly, antibody-mediated blockade of CTLA-4 during *P. berghei*-infection in ECM-resistant BALB/c mice resulted in higher levels of T cell activation, enhanced IFN-γ production, increased intravascular arrest of both parasitised erythrocytes and CD8^+^ T cells to the brain, and augmented incidence of ECM (36). Given that CD8^+^ T cells facilitate cerebral disease, CTLA-4 function appears to be protective against CM, as it prevents immune-mediated pathology by restricting T cell activation (54, 56).

### Inducible T Cell COStimulator (ICOS)

ICOS (CD278) is a CD28 homolog which regulates CD4^+^ T cell activation and promotes the induction of CD4^+^ follicular Th (T_FH_) cells which support B cell affinity maturation within germinal centers, to generate high-affinity antibodies. When ICOS deficient mice were infected with *P. chabaudi*, primary parasitemia was significantly lower compared to control mice with a corresponding higher frequency of Th1 cells during this early phase of infection (57). CD4^+^ T cells were capable of expressing PD-1, B cell lymphoma 6, and CXCR5 during early infection, indicating T_FH_ development was not impaired (57). However, capacity to control the chronic phase of infection, which is controlled by CD8^+^ T cells (18), was impaired without ICOS expression (57) indicating a role for ICOS in CD8^+^ T cell functions. Anti-ICOS treatment which depleted ICOS expressing CD4^+^ and CD8^+^ T cells during a *P. berghei* infection resulted in a concurrent reduction in plasma IFN-γ, confirming the influence of ICOS on Th1 responses (58). Further, although ICOS deficient mice could produce similar titres of parasite-specific antibodies of most IgG isotypes, the affinity of these antibodies was much lower than that of WT mice (57). This study showed that ICOS expression has a deleterious effect on protective Th1 immunity against malaria but is necessary for maintenance of a sustained high-affinity, protective antibody responses. This was confirmed by another study where ICOS expression by CD4^+^ T-cells, was limited by Interferon-alpha/beta receptor alpha chain (IFNAR1)-signaling to conventional DCs during *P. chabaudi* AS and *P. yoelii* 17XNL infections resulting in hindered resolution of infections, and impaired antibody responses (59).

ICOS expression by T cells may also be involved in the pathogenesis of cerebral malaria as indicated by studies with *P. berghei* which causes ECM in mice. Following *P. berghei* infection, mice with a deletion in the Fc receptor, FcγRIIB, or transgenic mice overexpressing toll like receptor 7 (TLR-7) had lower levels of cerebral pathology than WT mice, but were unable to control parasitemia (60). T cells from FcγRIIB deficient mice had uniformly intermediate levels of ICOS expression, while WT animals had two populations consisting of ICOS-high and–low T cells with the former being associated with better IFN-γ responses. This study indicated that ICOS plays a part in modulating Th1 immunity which supports cerebral pathology in malaria.

### HVEM/ /BTLA

The B and T lymphocyte attenuator (BTLA; CD272) and tumor necrosis factor superfamily member 14 (TNFSF14; also known as LIGHT; CD258) compete for interaction with herpesvirus entry mediator (HVEM; also known as tumor necrosis factor receptor superfamily member 14; CD270) and form a part of a complex family of co-signaling molecules. Ligation of LIGHT by HVEM is costimulatory, while BTLA-HVEM binding is co-inhibitory (61, 62). HVEM is expressed by many cells (e.g., hematopoietic, endothelial and epithelial cells), while the LIGHT is expressed on both innate and adaptive immune cells, and BTLA is expressed on naïve T and B cells and is further upregulated on activation.

Following infection of mice with cerebral malaria-causing *P. berghei*, both CD4^+^ and CD8^+^ T cells in the spleen exhibited increased BTLA expression (63). Agonizing this inhibitory receptor with an antibody reduced T cell infiltration in the brain. The T cells that did infiltrate the brain were less activated and produced less inflammatory cytokines. This effect of BTLA on cerebral pathology was only observed if the antibody was administered within the first 2 days of infection, indicating that the effect was due to reduced T cell priming. In contrast, BTLA knockout mice infected with non-lethal *P. yoelii*, exhibited strongly reduced parasitemia and cleared the infection earlier compared with wild-type mice (64). Protection was associated increased pro-inflammatory cytokine production by T cells, without the previously observed pathology (64). Thus, BTLA-HVEM interaction is relevant to malaria but while blockade would be beneficial to controlling parasitemia, it could increase the incidence of cerebral malaria. Of note, genetic variants of BTLA in humans, influence susceptibility to severe chronic hepatitis B (65), which could influence susceptibility to cerebral malaria.

Selective blocking of LIGHT and HVEM signaling does not protect mice from *P. berghei* ECM (66). However, LIGHT also binds to lymphotoxin β receptor (LTβR) besides HVEM (67). Blockade of LIGHT and LTβR signaling pathway early during *P. berghei* infection lead to increased splenic monocytes/macrophages, while blocking later in infection leads to reduced systemic cytokines, thus protecting mice from ECM (66). Thus, indicating that both BTLA and LIGHT are important immune regulators during experimental malaria.

### GITR

GITR is another member of the TNFR superfamily (TNFRSF) (68). GITR is expressed in lymphoid tissues and is involved in controlling activated T cells. While resting T cells express low levels of GITR that increase upon activation, CD4^+^CD25^+^ Treg cells express high levels (69). Agonist antibody against GITR enhances anti-CD3-induced proliferation of T lymphocytes, induce proliferation in anergic CD4^+^CD25^+^ Tregs and enhances proliferation of CD4^+^CD25^−^ responder T lymphocytes (70).

Multiple field studies have also implicated Tregs in the pathogenesis of malaria. Specifically, surface expression of GITR and intracellular expression of CTLA-4 were significantly upregulated in Tregs from *P. vivax*-infected individuals, with a positive association between either absolute numbers of CD4^+^CD25^+^FoxP3^+^GITR^+^ or CD4^+^CD25^+^FoxP3^+^CTLA-4^+^ and parasite load (71).

A role for Treg cells in mediating lethality of malaria was shown when mice survived lethal *P. yoelii XL* infections following depletion of Tregs (72). These Tregs were shown to inhibit the activation of effector T cells. However, Tregs did not affect the course of non-lethal *P. yoelii* 17XNL infections (72). Given that GITR is highly expressed by Tregs and supports their suppressive functions, an agonist antibody against GITR was tested during lethal and non-lethal malaria. However, GITR-signaling did not suppress Tregs during the lethal *P. yoelii* XL infection, indicating that Tregs developed a GITR-resistant mechanism during this infection (73).

In contrast, anti-GITR antibodies could partially reverse Treg-functions in *P. berghei* infections (74). Mice infected with *P. berghei* were shown to develop γδ T cells which secrete inflammatory cytokines IFN-γ and IL-17, but Tregs suppress expansion and abolish the effector function of antigen-activated γδ T cells (74). This suppressive effect of Tregs, could be partially reversed by using a monoclonal antibody to GITR. Overall, these studies suggest GITR may control only a subset of Tregs during malaria.

### OX40

Tumor necrosis factor receptor superfamily, member 4 (TNFRSF4 or CD134), also known as OX40 receptor, is a co-stimulatory receptor expressed on activated CD4^+^ and CD8^+^ T cells as well as a number of other lymphoid and non-lymphoid cells. The ligand for OX40, OX40L, is upregulated on APC following activation. Signals to OX40 promote expansion and survival of antigen-specific T cells (75).

Malaria patients and *Plasmodium*-infected rodents express OX40 predominantly on CD4^+^ T cells. Treatment of mice with an agonistic antibody against OX40 enhances helper CD4^+^ T cell activity, humoral immunity, and parasite clearance in during non-lethal malarial infections (76). OX40, is also upregulated on CD4^+^ and CD8^+^ T cells in the brain vasculature during ECM (77). Thus, while targeting OX40 may seems to be an attractive means to improve malarial immunity, previous studies showed improving immunity exacerbates ECM (36). Furthermore, agonizing OX40 and blocking PD-L1 during malaria without the complication of ECM, caused excessive IFN-γ responses which inhibited Tfh development required to clear the parasite (76). However, ligation of OX40 during non-lethal *P. yoelii* infection can increase the number of parasite-specific Th1-like memory cells which also exhibit phenotypic and functional features of Tfh cells during recall responses (78). Similarly, targeting OX40 with antibodies in conjunction with vaccination can also improve expansion of antigen-experienced effector CD8^+^ and CD4^+^ T cells and commitment protection (79). Overall, OX40 has a complicated function requiring further investigation, especially in the context of malaria.

### TIM3

TIM3 (also known as Hepatitis A virus cellular receptor 2), is expressed on CD4^+^ Th1 and CD8^+^ T cells. Galectin-9 was suggested to be a ligand for TIM3 (80) although this has been disputed for human T cells (81). TIM3 also binds phosphatidylserine (82). Ligation of TIM3 results in a selective loss of IFN-γ-producing cells and suppression of Th1-mediated autoimmunity (80).

TIM3 expression is significantly increased on a variety of T cells from *P. falciparum*-infected patients (83, 84). There are two main types of γδ T cells found in human blood, Vdelta1 and Vdelta2, and numbers of the latter increase significantly following *P. falciparum* infection in naive adults, but are lost in children following repeated exposure to malaria (83). The secretion of pro-inflammatory cytokines by these T cells was inversely associated with parasitemia and expression of TIM3 (73). Similarly, TIM3 expression is also significantly increased in mice infected with *P. berghei* (84, 85). Experimental models have shown upregulation of TIM3 during malaria leads to lymphocyte exhaustion which can be reversed with blockade of TIM3, resulting in the accelerated clearance of parasites and relief from the symptoms of *P. berghi*–mediated ECM (84). The role of TIM3 appears to be significant, requiring further investigation.

### CD40

CD40 is a costimulatory protein found on APCs, which is required for co-stimulation via CD154 (CD40L) on T cells. As CD40 is an essential component of Th1 and humoral immune responses, studies have looked for polymorphisms which may explain differences in susceptibility to malaria. Field studies in Mali have revealed a marginal susceptibility effect for the CD40L+220C allele from life-threatening malaria (86) but no effect on susceptibility to *P. vivax* in Brazil (87). However, from a functional viewpoint, γδT cells are essential for clearance of the malarial parasites as they have key role in dendritic cell activation via CD40 ligand expression and IFN-γ production (88, 89). As agonistic antibodies to improve immunity are being developed for the treatment of cancer, these could play a role in the future.

## Conclusion

Malaria is a complex disease and the failure to produce an efficacious vaccine indicates immune suppression mechanisms are impeding protection. The large number of studies cited in this review confirm an important role for immune checkpoint proteins in the pathogenesis malaria. However, the exceptional cost and potential side effects know to occur with immune checkpoint cancer treatments, as well potential exacerbation of cerebral malaria, has impeded any attempt to progress such treatments for malaria. Nevertheless, given rising drug resistance, difficulties in generating an efficacious vaccine for malaria and that some treatments such as soluble PD-L2 generate long-term protection with a reduction in the incidence of cerebral malaria, indicate that checkpoint blockade needs to be further explored.

## Author Contributions

All authors listed have made a substantial, direct and intellectual contribution to the work, and approved it for publication.

### Conflict of Interest Statement

The authors declare that the research was conducted in the absence of any commercial or financial relationships that could be construed as a potential conflict of interest.
